# Phenolic Azobenzene as Ligand for Cation Complexation—Syntheses and Applications

**DOI:** 10.3390/molecules30122499

**Published:** 2025-06-06

**Authors:** Jan Hočevar, Jernej Iskra, Estelle Leonard

**Affiliations:** 1 University of Ljublana, Faculty of Chemistry and Chemical Technology, Večna pot 113, 1000 Ljubljana, Sloveniajernej.iskra@fkkt.uni-lj.si (J.I.); 2 Université de Technologie de Compiègne, ESCOM, TIMR, F-60200 Compiègne, France

**Keywords:** phenol, azobenzene, photoisomerization, colorimetric sensors, DSSC dyes, cation complexes

## Abstract

Phenolic azobenzenes have garnered significant attention as functional materials due to their ability to undergo reversible photoisomerization and their potential for cation complexation. This review aims to provide a comprehensive overview of the recent developments in the synthesis, properties, and applications of phenolic azobenzene derivatives in cation binding and complexation. This article explores various synthetic strategies for the preparation of phenolic azobenzenes. Additionally, the mechanisms of cation complexation, including the role of the phenolic hydroxyl group and the azobenzene scaffold, are discussed, along with insights into the coordination chemistry involved. This review further examines the diverse applications of phenolic azobenzene complexes in fields such as ion sensing, catalysis, and biological and DSSC applications.

## 1. Introduction

### 1.1. General Presentation of Azobenzenes and Phenolic Compounds

#### 1.1.1. Azobenzene Properties

**Figure 1:**
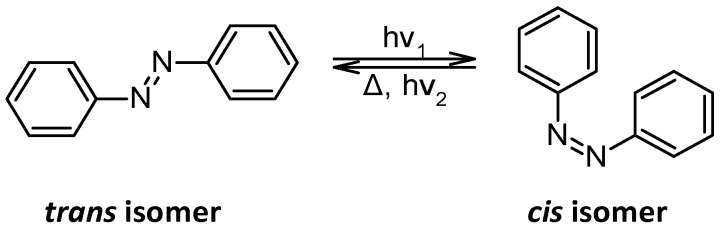
Two isomeric forms of azobenzenes.

Azobenzenes are organic molecules containing an azo (-N=N-) functional group linked to two benzene rings [1]. Their unique chemistry arises from their conjugated π-electron system, which affects their optical, electronic, and structural behaviour. One key chemical property of azobenzenes includes the fact that they exist in two isomeric forms, *trans* (E) and *cis* (Z), as shown in Figure 1.

**Figure 2:**
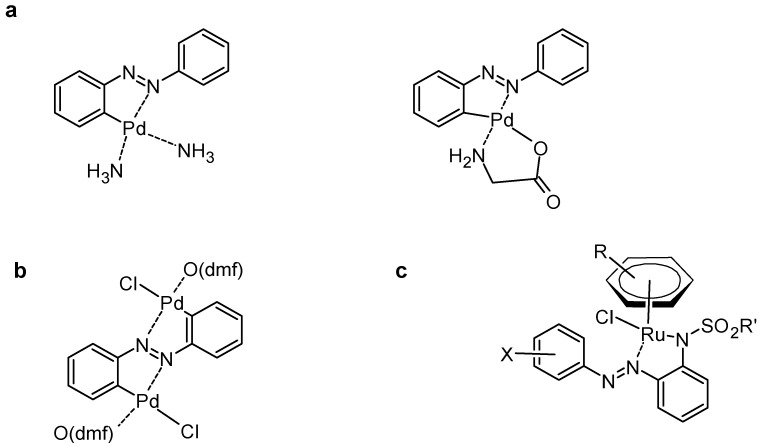
Examples of azobenzene metal complexes from (**a**) [11], (**b**) [12], and (**c**) [13].

The *trans* form is thermodynamically more stable, whereas the *cis* form has a higher energy and can revert to *trans* under thermal conditions [2]. The isomerization kinetics depend on substituents and solvent polarity. For example, electron-donating groups (-OH, -OCH_3_) lower the *cis*-to-*trans* activation barrier [3]. They also affect the electronic distribution. Indeed, electron-donating groups (EDGs) (e.g., -OH, -NH_2_) push electron density towards the azo bond, stabilizing the *trans* form and shifting absorption maxima to longer wavelengths (bathochromic shift). However, electron-withdrawing groups (EWGs) (e.g., -NO_2_, -CF_3_) pull electron density away, destabilizing the π-system and affecting light absorption properties [4]. Azobenzenes can undergo redox reactions [5], particularly one-electron reduction, forming radical intermediates. This reduction typically converts the azo (-N=N-) bond into a hydrazo (-NH-NH-) linkage [6,7,8,9] under acidic or catalytic conditions. Azobenzenes act as ligands in metal complexes, coordinating with transition metals such as Fe, Ru, and Pd. Their ability to form charge transfer complexes makes them valuable in organometallic catalysis [10]. For example (Figure 2), in 2016, cyclometallated azobenzene palladium(II) complexes where synthesized, characterized using electronic absorption spectroscopy and electrochemical studies [11]. In 2008, dicyclopalladated complexes were synthesized using azobenzene derivatives, where the palladium centres form five-membered chelate rings through orthometallation. These complexes were characterized by X-ray crystallography and computational methods, providing insights into their structural and electronic properties [12]. A series of arene ruthenium complexes containing o-sulfonamide azobenzene ligands was also synthesized. These complexes exhibited uncommon coordination patterns with an exocyclic N=N bond. Upon irradiation, they underwent *trans* → *cis* photoisomerization reversibly by thermal isomerization, with rates dependent on solvent, arene group, sulfonamide moiety, and azobenzene substitution [13].

#### 1.1.2. Phenolic Properties

**Figure 3:**
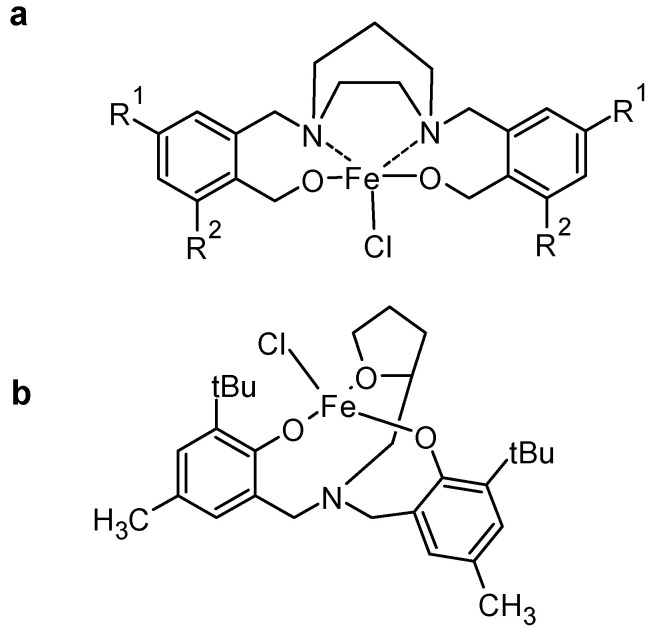
Phenolate metal coordinates from (**a**) ref [18] and (**b**) ref [19].

Phenolic compounds are aromatic molecules featuring one or more hydroxyl (-OH) groups attached to a benzene ring. Their chemical reactivity is largely determined by the electron-donating effects of the hydroxyl group and its ability to participate in various chemical transformations. Phenols are weak acids (pKa~9–11) and can donate protons to form phenoxide anions in basic solutions. The phenoxide ion (-O^−^) is more reactive than its neutral form. Phenols can be easily oxidized to quinones [14,15] (e.g., hydroquinone to benzoquinone), and such transformations are crucial in redox reactions, dye chemistry, and electron transport mechanisms. The oxidation rate depends on substituents and environmental pH. The hydroxyl group in phenols participates in hydrogen bonding [16], influencing solubility and physical data (melting and boiling points, for example). Strong hydrogen bonds make phenols more soluble in polar solvents. Phenolic -OH groups can coordinate with metal ions (Fe^3+^, Cu^2+^) [17], forming coloured metal complexes. For example, Dalal Alhashmialameer and colleagues synthesized a series of iron(III) complexes supported by tetradentate amino-bis(phenolate) ligands. These complexes exhibit square pyramidal geometries, as confirmed by X-ray crystallography. They have been studied as catalysts for the formation of organic carbonates from CO_2_ and epoxides. The Fe–O(phenolate) bond lengths range from approximately 1.8690(12) to 1.8805(12) Å, indicating strong iron–oxygen interactions [18]. Another example is detailed by Christopher M. Kozak and co-workers, who synthesized six new iron(III) diamine-bis(phenolate) complexes. These structures were elucidated through single-crystal X-ray diffraction (Figure 3) [19].

Finally, the main properties are summarized in (Table 1).

### 1.2. Objectives and Scope of This Review

This review focuses on phenolic azobenzenes, highlighting their reversible photoisomerization behaviour and utility in cation complexation. It aims to present a comprehensive summary of recent advancements in their synthesis, structural features, and functional properties. This review explores diverse synthetic methodologies, emphasizing the role of the phenolic hydroxyl group and azobenzene scaffold in coordinating cations. Mechanistic insights into complexation processes are examined alongside their relevance to coordination chemistry. Furthermore, this review outlines the key applications of phenolic azobenzene–cation complexes, particularly in ion sensing, catalysis, biological systems, and dye-sensitized solar cells (DSSCs), thereby defining their significance as multifunctional materials.

## 2. Chemistry of Phenolic Azobenzenes

### 2.1. Structure and General Properties of Hydroxyazobenzenes

Hydroxyl-azobenzenes are organic compounds featuring both hydroxyl (-OH) and azo (-N=N-) functional groups attached to benzene rings. The position of the hydroxyl group significantly influences their properties. In *para*-hydroxyazobenzene, for example, ground-state keto/enol tautomerism is observed, affecting its photochemical behaviour [20]. Upon excitation, the *trans*-keto form can isomerize to the *cis* configuration, with the tautomeric equilibrium and isomerization dynamics being solvent-dependent. In aqueous solutions, *para*-hydroxyazobenzene exhibits considerable keto/enol tautomerism in the ground state, leading to specific excited-state behaviours. Indeed, the excited-state intramolecular proton transfer (ESIPT) in *para*-hydroxyazobenzene involves a photoinduced tautomerization between azophenol and quinone-hydrazone forms. Upon UV excitation, the molecule can undergo an ultrarapid intramolecular proton transfer. In the ground state, p-HAB predominantly exists in the enol form, stabilized by intramolecular hydrogen bonding. Upon excitation, the proton transfer to the azo nitrogen occurs, forming the keto tautomer in the excited state [21]. The ESIPT process in p-HAB is influenced by solvent polarity and pH. Polar solvents and acidic conditions can stabilize the keto form, enhancing the ESIPT process. Conversely, in nonpolar solvents or under basic conditions, the enol form is favoured, and ESIPT is less prominent [22].

These characteristics make hydroxyazobenzenes valuable in applications such as light-driven optical switching and photoactive monomers in elastomers for light-sensitive actuators [23]. The infrared (IR) spectra of hydroxyazobenzene compounds [24] exhibit characteristic absorption bands corresponding to different functional groups, including hydroxyl (-OH), azo (-N=N-), and aromatic (-C=C-) vibrations. The characteristic (N=N) IR absorption bands for hydroxyazobenzene are typically observed around 1400–1450 cm^−1^, but these bands can shift depending on substituents and conjugation with the benzene rings. Other bands (OH, aromatic CH, and CO) are usually observed. However, the exact peak positions and intensities may vary depending on the substituents present on the benzene rings and whether the hydroxyl group participates in intramolecular hydrogen bonding (e.g., with the azo group). Moreover, in the case of *o*-hydroxyazobenzene, it has been established by polarized IR spectra in the region of 4000–400 cm^−1^ that the molecule of 4-chloro-2′-hydroxy-4′-pentyloxyazobenzene undergoes a spontaneous ordering in thin layers of 10–20 μm [25]. The broad absorption in this region can be attributed to plane-to-plane intermolecular interactions, driven by resonance-assisted hydrogen bonding (RAHB) involving intramolecular O–H⋯N hydrogen bonds [26].

**Figure 4:**
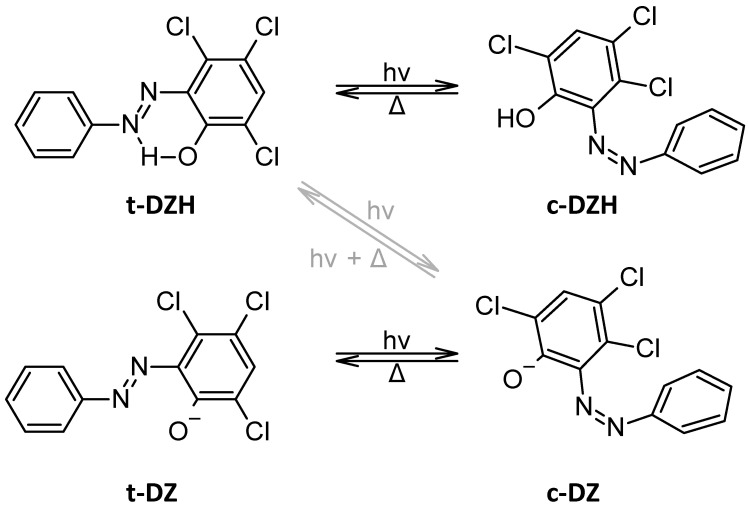
Molecular structures of t-DZH, c-DZH, t-DZ, and c-DZ with classical *trans* → *cis* isomerization (black arrows, [27]) or light-driven ionization (grey arrows, [28]).

The UV–visible spectroscopy of hydroxyazobenzenes is a crucial technique for studying their electronic properties and photochemical behaviour. These compounds typically exhibit strong absorption in the visible region due to the electronic π-π* transition of the conjugated azo (-N=N-) system, often accompanied by a weaker n-π* transition. The presence of a hydroxyl (-OH) group at the *ortho* or *para* position to the azo group significantly influences the absorption spectra due to mesomeric effects and intramolecular interactions, particularly hydrogen bonding. In acidic or basic media, these compounds can undergo bathochromic or hypsochromic shifts due to changes in electron density and tautomeric equilibrium (equilibrium between the azo and hydrazone forms). These properties make hydroxyazobenzenes particularly interesting for applications as dyes, pH indicators, or photochromic materials. For example, (E)-3,4,6-trichloro-2-(p-diazenil)-phenol (t-DZH) can be deprotonated in t-DZ (Figure 4), and this transformation has a strong impact on *trans* → *cis* isomerization [27]. Moreover, these hydroxyazobenzenes can also reversibly photogenerate tuneable pH drops in water up to one pH unit amplitude upon illumination at 365 nm (Figure 4, grey arrows, [28]).

### 2.2. Solvatochromism and Complexation Effects of Azo–Schiff Bases and Hydroxyl-Azobenzenes

**Figure 5:**
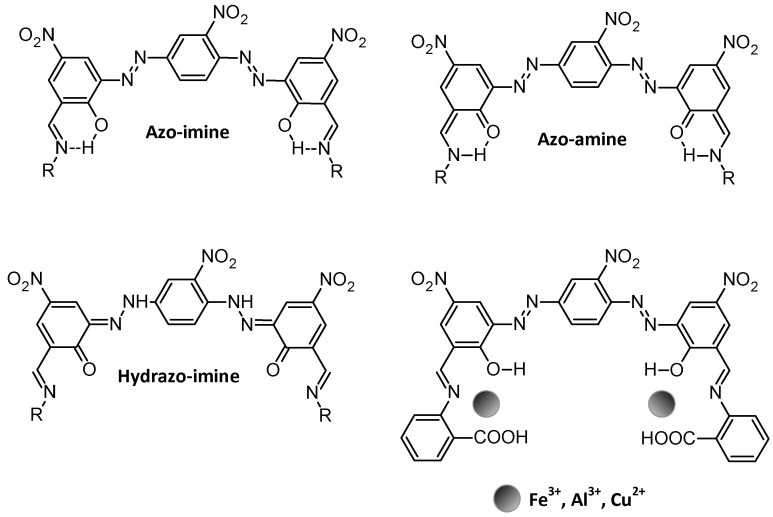
Proposed tautomerism and binding interaction of azo–Schiff base [32].

Phenolic azo–Schiff bases are a specialized class of ligands that incorporate phenolic, azo, and imine (-C=N-) functional groups, offering multiple coordination sites for metal cation complexation [29]. The phenolic OH group, in particular, plays a crucial role by donating electron density to metal centres, often enhancing complex stability through hydrogen bonding or deprotonation to form phenolate ions [30]. This multi-dentate coordination capability allows phenolic azo–Schiff bases to form stable chelate complexes with a wide range of transition metal ions [31]. While azo dyes exist in azo or hydrazone tautomeric forms, 2-hydroxy Schiff bases exist in phenol-imine, hydrazo-imine, or keto-amine tautomers in solid state and solution. When complexation occurs with metal ions, tautomerization favours the phenol-imine isomer (Figure 5) [32].

## 3. General Synthetic Methods for Azobenzenes and Hydroxyazobenzenes

**Figure 6:**
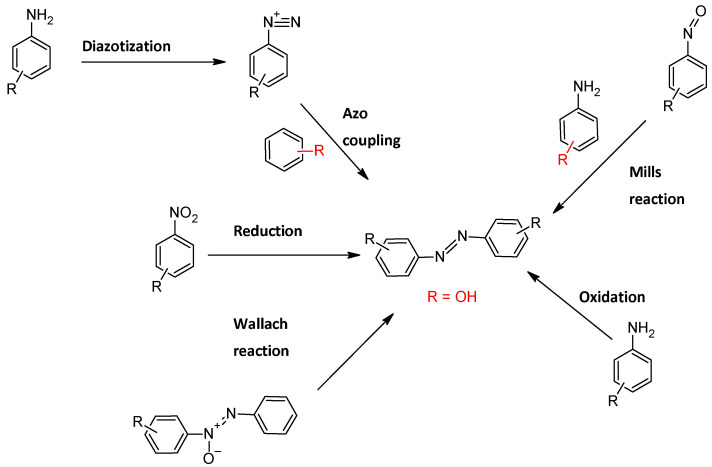
General synthetic methods for synthesis of azobenzenes.

Various synthetic strategies have been developed for the preparation of phenolic azobenzenes, each with specific advantages in terms of selectivity, efficiency, and compatibility with functional groups [33]. These methods usually involve either the coupling of phenolic compounds with azobenzene derivatives or the direct formation of the azo bond between anilines and phenols under controlled conditions [34]. The most commonly used methods for the synthesis of azobenzenes include the azo coupling reaction, the Mills reaction, the Wallach reaction, the oxidation of anilines, and the reduction of nitroaromatic compounds (Figure 6) [35]. While synthetic methods for symmetrically substituted azobenzene derivatives are well established, the development of efficient strategies for their asymmetrical counterparts remains an ongoing challenge [36].

### 3.1. Azo Coupling Reaction

**Figure 7:**
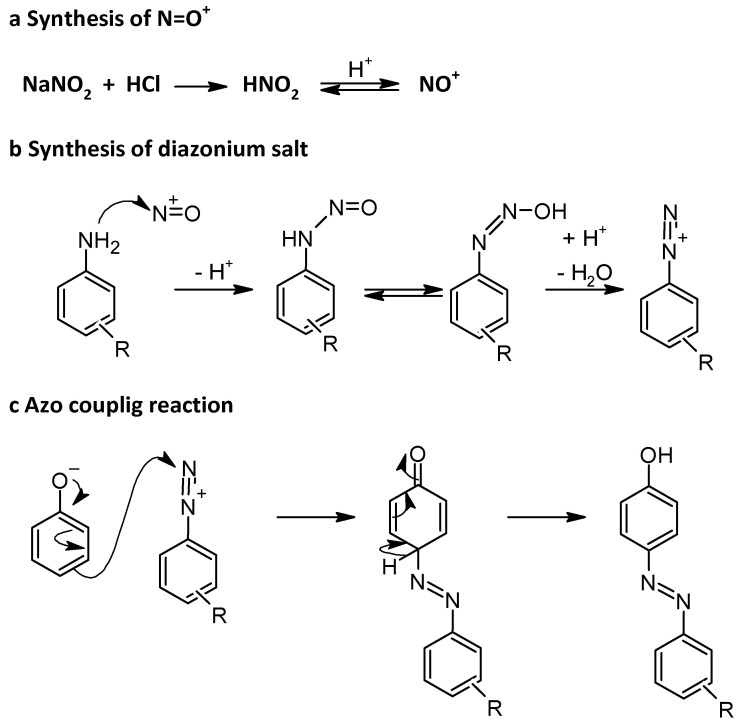
Mechanism of azo coupling reaction.

Azo coupling reactions are among the most commonly used methods for the synthesis of azobenzenes, involving an electrophilic aromatic substitution (S_E_Ar) reaction of a diazonium salt and an electron-rich arene [37]. The methodology usually begins with the diazotization of an aromatic primary amine using an in situ prepared nitrosonium ion, which is carried out under mild conditions at low temperature (0 °C to 5 °C) to form the diazonium salt (Figure 7) [38]. The electrophilicity of diazonium ions is relatively weak because their positive charge is delocalized. Therefore, phenols as electron-rich arenes are suitable substrates for the coupling reaction with aryl diazonium salts to form the azo compound. 

**Figure 8:**
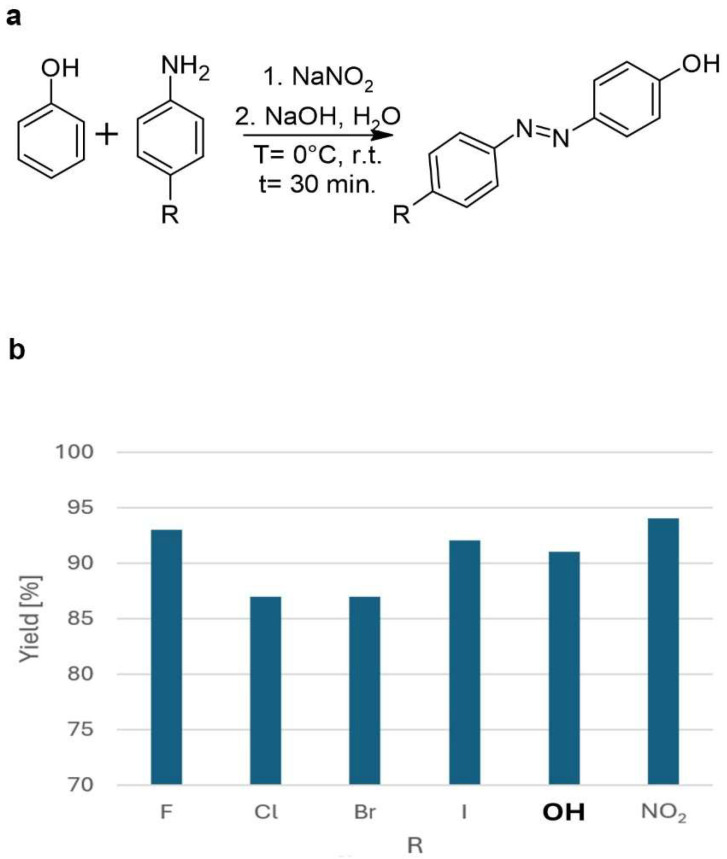
Yield of azobenzenes in coupling reaction of phenol with substituted anilines. (**a**) General pathway and (**b**) yields depending on R.

The substitution usually takes place at the para position, unless this is already occupied, in which case the ortho position is preferred. The reaction often requires short reaction times and generally leads to products of high purity (Figure 8) [39]. This process is extremely versatile and allows for the selective functionalization of both the amine and the nucleophilic component, making it suitable for a wide range of substrates (Figure 8) [40].

As can be seen from the diagram in Figure 8, the yield of azobenzene formation does not depend on the electronic properties of the substituents, and high yields of azobenzene products of 85% to 95% were obtained, irrespective of whether the substituent on the aniline is electron-withdrawing or electron-donating. However, this method requires careful temperature and pH control to ensure the stability of the diazonium salt and to avoid side reactions [41]. The side reactions related to azo coupling are mainly the decomposition of diazonium salt to phenols, chlorobenzene (if hydrochloric acid was used to form this salt), or to any functionalized benzene depending on the presence of nucleophilic moieties [42]. Moreover, the use of sodium nitrite can lead to the formation of nitrobenzene from the corresponding aniline [43]. This is why the acid and temperature used in diazotization not only adjust the pH of the reaction medium to slightly acidic or neutral conditions but also play a crucial role in determining the stability of the resulting diazonium salt. Diazonium chloride salts, traditionally prepared from aniline, sodium nitrite, and hydrochloric acid, are unstable at room temperature and are normally prepared and used at 0–5 °C. However, diazonium compounds can be isolated as tetrafluoroborate or tosylate salts, which form stable solids at room temperature [44].

In the case of not performing the in situ conversion of aniline to azobenzene, it is also possible to isolate the diazonium salt, which can be further converted to the phenolic azobenzene via a reaction with a sodium acetate (NaOAc) base in aqueous medium (Table 2) [45].

As can be seen in Table 2, the use of a polar solvent is key to achieving higher conversion efficiencies. In fact, when a higher proportion of water is used, the efficiency ranges between 80% and 90% [45].

### 3.2. Baeyer–Mills Reaction

**Figure 9:**
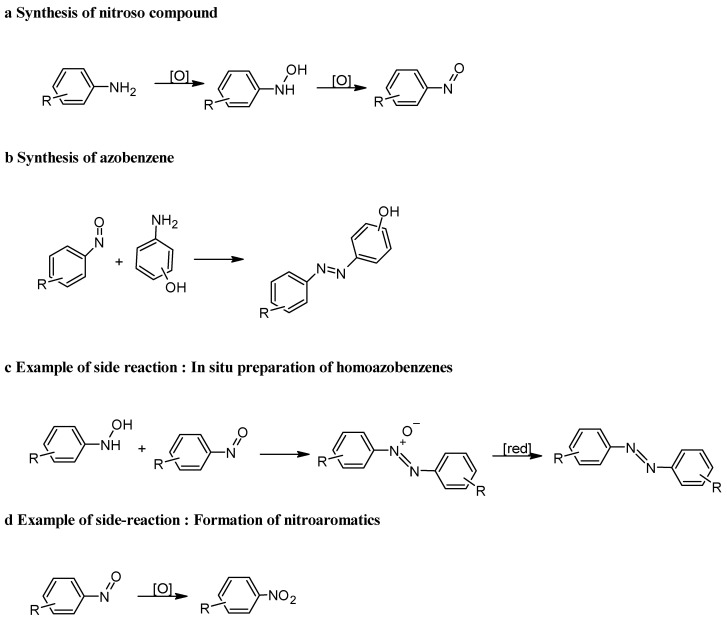
Mechanism of Baeyer–Mills reaction.

The Baeyer–Mills reaction is one of the most commonly used methods for accessing non-symmetric azobenzenes and is based on the condensation of nitrosobenzene derivatives with anilines [46]. This reactivity can be explained by the proposed mechanism, which involves the nucleophilic attack of the aniline on the nitrosobenzene derivatives in acidic or basic media (Figure 9) [47,48]. The Baeyer–Mills reaction consists of several steps. In the first step (Figure 9a), aniline is oxidized to a nitroso compound, whereby *N*-phenylhydroxylamine is formed as an intermediate. In the next step (Figure 9b), a reaction takes place between the nitroso compound and aniline, producing azobenzene. The use of suitable reagents and appropriate catalysts is crucial for this reaction, as otherwise, symmetrical azobenzenes are formed in situ (Figure 9c) or overoxidation takes place, leading to the formation of nitroaromatics (Figure 9d) [49].

The reaction exhibits good selectivity, with control over the electronic properties of the reactants, allowing for the fine-tuning of the final product [46]. The presence of electron-donating groups, such as hydroxyl groups, on the aniline ring can further influence the reactivity and regioselectivity of the process, making this method highly adaptable for the synthesis of functionalized phenolic azobenzenes (Table 3) [50]. Table 3 shows that electron-rich anilines perform poorly in Baeyer–Mills reactions because they have a high propensity to reduce nitrosoarene, which translates into higher yields of the azoxybenzene side products. Anilines with moderately electron-withdrawing and electron-donating substituents give excellent yields of the desired azobenzenes with a minimal formation of azoxybenzene. The yield of azobenzene decreases sharply when arylamines with strongly electron-withdrawing substituents are used, which is probably due to the lower nucleophilicity of the anilines [20]. The position of the functional group in relation to the amine group also affects the yield of azobenzene synthesis. The data presented in Table 3 show that the conversion of aniline (regardless of the nature of the substituent) in the Baeyer–Mills reaction is quite high, with the exception of the 2,6-difluoro and nitro functional groups, where the conversion rate is between 10% and 25%.

In cases where azoxybenzene is formed as the main product of the Baeyer–Mills reaction, the subsequent reduction of azoxybenzenes to azobenzenes occurs, and this is discussed in the following sections of this paper.

**Figure 10:**
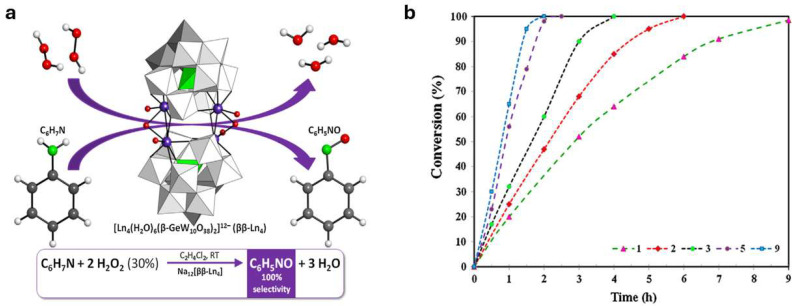
(**a**) Aniline-to-nitrosobenzene selective oxidation process and molecular structure of Na-ßß -Ln_4_ catalysts and (**b**) aniline conversion at room temperature throughout nine consecutive runs. Reprinted with permission from Ref. [51]. Copyright 2015, Elsevier Inc.

As can be seen from the above figures and the table, the key to the Baeyer–Mills reaction lies in the production of the nitroso compound. Generally, this is produced by the oxidation of aniline, but it should be noted that not all oxidation conditions allow for the production of a nitroso compound (Figure 10). The nitroso compound can be formed either with Oxone, which is also the most common process, or with another oxidizing agent such as hydrogen peroxide in the presence of a suitable catalyst [51]. Although H_2_O_2_ presents major problems in transport and handling, it is a green oxidizing agent, due to the formation of non-toxic and environmentally benign byproducts, besides presenting an excellent atom efficiency. In principle, Oxone provides better selectivity for the formation of nitroso compounds, since in the case of the use of H_2_O_2_/catalyst (using classical catalysts such as SeO_2_, Na_2_WO_4_, MTO), azoxybenzene is often formed directly. On the other hand, new catalytic systems have been developed which, in combination with H_2_O_2_, allow for the highly selective conversion of aniline to nitroso compounds. One such catalyst is Na-ßß -Ln_4_ (Figure 10a), a dimeric POM (polyoxometalate) composed of lanthanide-containing dilacunary Keggin subunits of the β-type that act as Lewis acids, enabling the full conversion of the substrate and total selectivity towards nitrosobenzene. The exact nature of the lanthanide centres affects not only catalytic activity but also the solubility of the catalyst during the reaction. Reaction rates decrease as the atomic number of lanthanide increases, but at the same time, less of the catalyst is transferred to the solution.

Moreover, the catalyst has excellent recyclability properties. The selectivity of the catalyst towards nitrosobenzene remains constant (100%) even after nine consecutive reaction runs. Interestingly, the time required to obtain 100% conversion decreases in the successive reactions (Figure 10b) [51].

**Figure 11:**
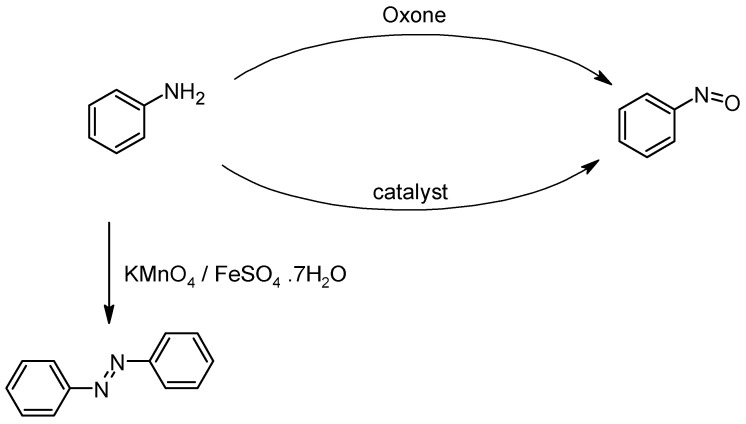
Oxidation of aniline to nitroso derivative or symmetrical azobenzenes using suitable oxidants.

When a combination of KMnO_4_ and FeSO_4_ is used as an oxidizing agent (Figure 11), this reaction does not lead to the oxidation of aniline to nitrosobenzene. Instead, symmetrical azobenzene is formed directly [52,53].

With certain oxidizing agents, asymmetric azobenzenes can be formed directly and not via the synthesis of a nitroso intermediate [54].

In cases where nitroso compounds are not involved as intermediates in the reaction mechanism, the process can no longer be classified as a Baeyer–Mills reaction but rather as the oxidative coupling of anilines, leading to the formation of azobenzenes, as discussed in the following section of this review article.

### 3.3. Oxidation of Anilines

The essential difference between the Baeyer–Mills reaction mentioned above and the in situ formation of azobenzene by the oxidation of anilines is that in the latter case, the intermediate oxidized form of aniline is not isolated. It is precisely for this reason that symmetrical azobenzenes are formed in this type of synthesis.

The formation of azobenzenes by the oxidation of aniline is a fairly old process, originally based on electrolytic oxidation [53]. In general, these processes were characterized by synthesizing azobenzenes in lower yields than those of other processes. An example of electrolytic oxidation is the synthesis of azobenzene from trichloroaniline, which was carried out with a platinum (Pt) electrode in dimethylformamide and in the presence of pyridine [55].

It has been reported that various oxidizing agents can generate azo compounds from aromatic amines. When H_2_O_2_/Na_2_WO_4_ is used, the initial oxidation of aniline to the azodioxide and its subsequent reduction with Si_2_Cl_6_ take place. Azobenzene is obtained in very good yield [56].

A variety of metallic and non-metallic reagents, such as Ag_2_O, AgO [57], AgMnO_4_ [58], MnO_2_ [59], KO_2_ [60], NaBO_3_ [61], Pb(OAc)_4_ [62], BaMnO_4_ [63], Ce(OH)_3_O_2_H [64], NiO_2_, peroxides (such as hydrogen peroxide or *tert*-butyl hydroperoxide [65]), and even chemicals from electrochemical oxidation [66], were used either stoichiometrically or in excess to synthesize azobenzenes from aromatic amines. This literature review showed that the choice of oxidation reagent has a really big impact on the final yield of azobenzene synthesis. It turns out that the yields in the case of the use of specialties such as BaMnO_4_, MnO_2_, and AgMnO_4_ are high and range between 70% and 90%, in contrast to, for example, NaBO_3_, Ce(OH)_3_O_2_H, NiO_2_, and AgO, where the yields are mostly lower than 60%. Furthermore, it is also interesting to note that the yield is also affected by which manganese speciation is used as the oxidation reagent. For example, oxidation with barium manganate gives reproducible results without requiring further activation, while an oxidation reaction with manganese dioxide is not reproducible in many aspects. In addition to the above, BaMnO_4_ allows for higher selectivity in oxidation reactions, while at the same time, they are faster and have higher reaction yields [63].

Gold nanoparticles on titanium dioxide (TiO_2_) have also been shown to be a very efficient system for the conversion of aniline to azobenzenes, allowing for a very selective conversion to azobenzene (up to 98 %) [67].

Nowadays, the use of green oxidizing agents such as oxygen/air or hydrogen peroxide (H_2_O_2_) in particular is being researched for this type of oxidation [68], due to the formation of non-toxic and environmentally benign byproducts. In this case, oxidation reactions are carried out using suitable catalysts such as Zr(OH)_4_, and the azobenzene formation efficiencies are between 70% and 90%. However, when oxygen is used as the oxidation reagent, the yields increase above 90%, as a more selective formation of azobenzene occurs with a relatively low proportion of nitrosobenzene (around 2%) and is not significantly dependent on the functional groups present on the aniline [65].

Although the oxidation process is often efficient, it must be carefully controlled to avoid overoxidation, which leads to undesirable byproducts. For example, excessive oxidation can lead to the formation of azoxy or nitro compounds, which are usually more stable and may not be suitable for subsequent reactions or applications. Therefore, the optimization of reaction conditions, including the amount of oxidant and reaction time, is critical to achieve high yields of the desired phenolic azobenzene products. For example, in the case of NaBO_3_, the temperature of the reaction mixture proves to be very important. It turns out that the yield of the reaction increases with increasing temperature. In addition to the above, the pH of the reaction mixture also has a major influence, i.e., the yield of the azobenzene formation reaction decreases with an increasing pH of the reaction mixture. In the case of metal peroxides, it turns out that the reaction times in the case of nickel(II) peroxide are much longer than those in the case of ceric trihydroxy hydroperoxide. In the case of the use of O_2_ or peroxide as the oxidation reagent in combination with a catalyst, it turns out that reaction conditions such as reaction time and the concentration of the oxidation reagent do not significantly affect the yield of the azobenzene synthesis reaction itself. On the other hand, the choice of peroxide and solvent is very influential. It was found that only the reaction in acetic acid and in the presence of the oxidizing agent tert-butyl hydroperoxide is selective as far as the synthesis of azobenzenes is concerned. In the other cases (using H_2_O_2_ in combination with H_2_O, methanol, and mesitylene as solvents), a nitroso compound or an azoxybenzene is formed. If azoxybenzene is formed in the reaction, it can also be converted to azobenzene with a suitable reducing agent (e.g., Si_2_Cl_6_). In the case of using oxygen as an oxidizing agent, mesitylene proves to be a significantly better solvent than water in terms of the selectivity of azobenzene formation. On the other hand, the reaction time and solvent do not significantly affect the yield of the azobenzene formation reaction in the case of AgO.

**Figure 12:**
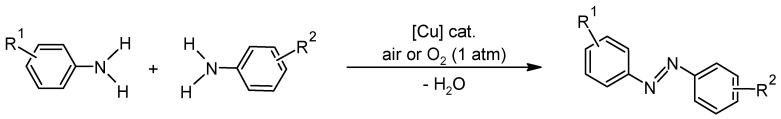
Oxidation of anilines to form asymmetrical azobenzene.

In contrast to the above oxidation processes, the synthesis of asymmetric azobenzene can be achieved in combination with air or oxygen at standard pressure (1 atm) and the use of a copper catalyst (e.g., CuBr or CuCl) (Figure 12 [54]). A similar reaction pathway can be observed in the case of manganese oxide using chlorobenzene as solvent, when in the presence of oxygen and heated to 160 °C [69].

The above method of the oxidation of anilines and the formation of azobenzenes also allows for the synthesis of symmetrical azobenzenes in yields ranging from 60% to 100% depending on the functional groups on the aniline. In the case of symmetrical azobenzene synthesis, the reaction yields are lower, ranging between 40% and 70% depending on the functional groups on the aniline.

### 3.4. Reduction Reaction of Azoxybenzenes

As shown in the previous sections, overoxidation produces azoxybenzenes containing a N=N bond with an additional oxygen functionality (-N=N(O)-), which can be removed by controlled reduction to give the corresponding azo compounds, leaving the conjugated π-system intact.

The first way to reduce azoxybenzenes is to use various reducing reagents, including hydrogenation catalysts (e.g., palladium or platinum on carbon), metal-based reducing agents (such as zinc, iron, or tin in acidic media), and organic reducing agents such as hydrazine or sodium dithionite [70,71,72]. In addition, the reduction of azoxybenzenes to azobenzenes can also take place via photochemical conversion [73]. Upon direct irradiation, azoxybenzenes may undergo rearrangement to 2-hydroxyazobenzene and *cis*-*trans* isomerization. In contrast, being irradiated in the presence of a photosensitizer (such as acetophenone, benzophenone, thioxanthone, flavone, fluorenone, etc.) with suitable triplet excitation energy leads to a reduction to azobenzene. In the absence of sensitizers, only a change in the geometric configuration of azoxybenzene occurs, and 2-hydroxyazobenzene is formed. As the concentration of the sensitizer increases, the proportion of azobenzene formed also increases. If oxygen is also present in the reaction, it causes a decrease in the proportion of azobenzene formed. The following ketones were efficient photosensitizers to be used to reduce azoxybenzenes to azobenzene: acetophenone (triplet excitation energy ET1 = 74 kcal/mol), benzaldehyde (72 kcal/mol), benzophenone (69 kcal/mol), thioxanthone (65 kcal/mol), phenylglyoxal (63 kcal/mol), and anthraquinone (62 kcal/mol). On the other hand, the following ketones were not efficient: flavone (62 kcal/mol), Michler’s ketone (61 kcal/mol), 2-acetonaphthone (59 kcal/mol), biacetyl (55 kcal/mol), benzil (54 kcal/mol), and fluorenone (53 kcal/mol). Based on these results, the triplet state energy of the azoxybenzene intermediate is estimated to be approximately 62 kcal/mol. It is proposed that 2-hydroxyazobenzene and *cis*-azoxybenzene originate from the excited singlet state of azoxybenzene, whereas azobenzene forms via the triplet excited state [73].

**Figure 13:**
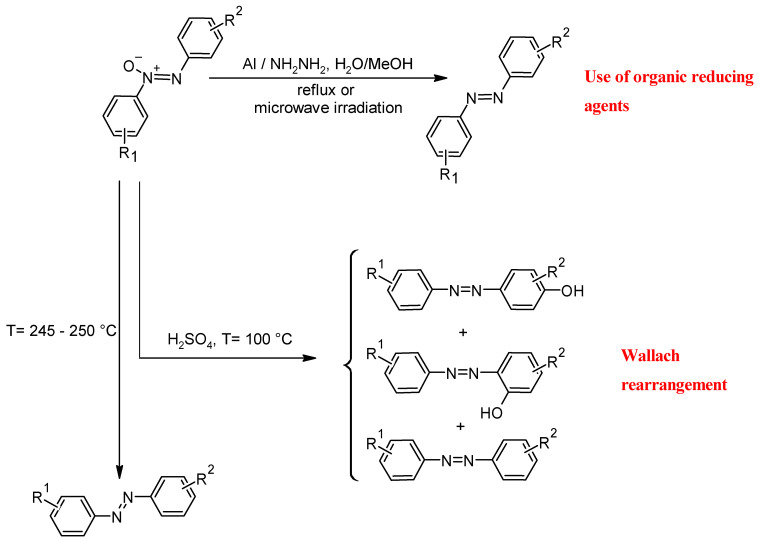
Reduction reaction of azoxybenzenes and Wallach rearrangement.

The reduction of azoxybenzene is usually stepwise, with the oxygen atom being removed to form the azo product. The choice of reducing agent and reaction conditions has a significant impact on the efficiency, selectivity, and functional group tolerance of the process [74]. A process in which azoxyarenes are treated with hydrazine hydrate in the presence of aluminum in methanol under reflux or microwave irradiation is commonly used for such reactions (Figure 13). In general, the yields are quite high in the conversion of azoxybenzenes to azobenzenes (between 85% and 100%), and they are independent of the type of heating of the reaction mixture. On the other hand, the kinetics of the reaction depend mainly on the type of heating. In the case of microwaves, the reaction time is only 2 min, whereas in the case of reflux, it is about five times longer [71].

**Figure 14:**
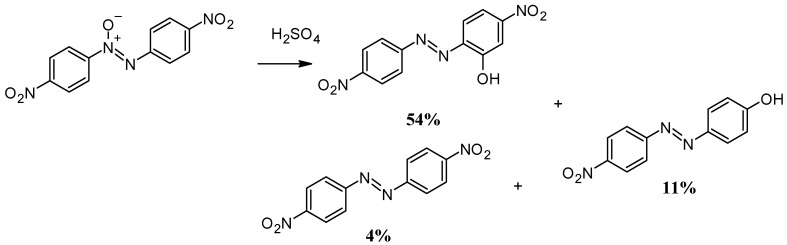
Wallach rearrangement in *para*-disubstituted azoxybenzene.

Another option for introducing a hydroxyl functional group into azobenzene is the Wallach rearrangement. In this reaction, the conversion of azoxybenzene to azobenzene occurs by the rearrangement of the hydroxy group to an azoxy functional group on the aromatic ring. Although the Wallach reaction is used less frequently than other synthesis methods, it is particularly suitable for the regioselective formation of phenolic azobenzenes [75]. Wallach rearrangement only takes place in strongly acidic solutions. It combines the loss of the oxygen bound to the azo group with the formation of a hydroxyl group in the para position to the azo bond in the benzene ring. In contrast to conventional azo coupling reactions based on diazonium salts or nitroso condensation, the Wallach reaction offers an alternative route to azobenzenes, especially in cases where the direct oxidation of aniline derivatives is difficult [76]. As shown in Figure 13, the main product of the Wallach rearrangement is the phenolic azobenzene, which has an -OH functional group at the para site. This rearrangement also produces byproducts such as *ortho*-hydroxyazobenzene, azobenzene, and some polymeric materials from aniline and sulfonic acids in very small amounts [77]. The reaction of unsubstituted azoxybenzene at room conditions yields 72% *p*-hydroxyazobenzene and 28% azobenzene. In the Wallach rearrangement, sulphuric acid is generally used in concentrations of over 80% after heating to T ≥ 50 °C. With increasing acidity, a slight increase in the amounts of azobenzene and polymeric substances is observed. When azoxyazobenzene is heated to higher temperatures (245–250 °C), azobenzene is formed as the main product with small amounts of *para*- and *ortho*-hydroxyazoxybenzene (Figure 13). In the case of Wallach rearrangement, the formation of a hydroxyl functional group can also promote the dehydration process, leading to the subsequent formation of the polymeric form of azobenzene [78]. In the case of unsubstituted azoxybenzene, 32% *p*-hydroxyazobenzene and 68% azobenzene are obtained. If the para position is occupied, a hydroxyl group forms in the ortho position (Figure 14) [79].

### 3.5. Reductive Coupling of Aromatic Nitro Derivatives

This reaction involves the selective reduction of nitroarenes to nitroso derivatives, which are starting substrates for the formation of azobenzene(-N=N-). The reaction leads to the production of symmetrically substituted azo derivatives [80]. Commonly used reducing agents include metal-based reagents such as zinc, iron, or tin in acidic or neutral media and catalytic hydrogenation with transition metal catalysts such as palladium, platinum, or ruthenium [81,82]. Nitroaromatics can also be reduced in the absence of a metal-based catalytic system using an alkaline medium or a combination of sodium hydroxide and ethanol [83]. In addition, glucose is often used as a reducing agent in alkaline media [84].

**Figure 15:**
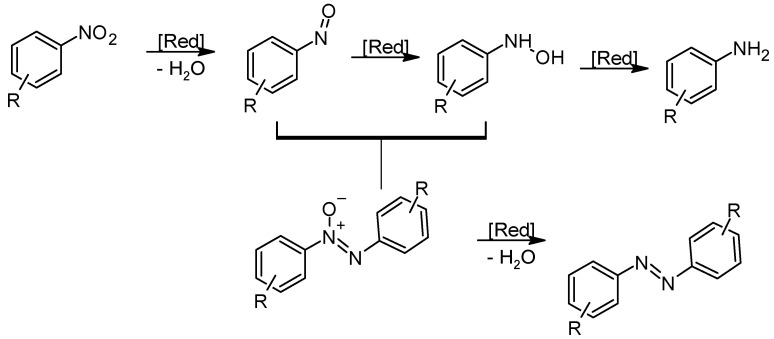
Reductive coupling of aromatic nitro derivatives.

The reaction proceeds via the stepwise reduction of the nitro group, producing reactive intermediates such as nitroso and hydroxylamine species. These intermediates are then coupled, leading to the formation of the azo bond (Figure 15). The reduction process is very sensitive to the choice of reducing agent, catalyst, and reaction conditions, which significantly influence the selectivity of the reaction [85]. Controlling these parameters is important to prevent over-reduction to aniline derivatives [86]. The choice of metal has a major influence on the yield of the reaction in the synthesis of symmetrical azobenzene. Lithium or zinc is most commonly used as a reducing agent in conjunction with an active metal such as Mg, Zn, Al, In, Ti, or Nb. It can be seen that when the metals W, Mo, and Zr as activated metal are used, the reaction to form azobenzene from nitroaromatics does not take place at all [87]. The functional groups present on the nitroaromatic compound also have a major influence on the yield of the reaction. It turns out that they are the most likely to lower the yield of the azobenzene synthesis reaction. One of the main advantages of this method is its versatility, as it is suitable for a wide range of nitroaromatic compounds, including those with electron-donating or electron-withdrawing substituents. The use of metal-based reducing agents provides an efficient route for the selective generation of azo compounds, while catalytic hydrogenation offers a mild alternative with excellent scalability. In addition, the process can be adjusted to control the regioselectivity and functional group compatibility of the resulting azobenzene products.

**Figure 16:**
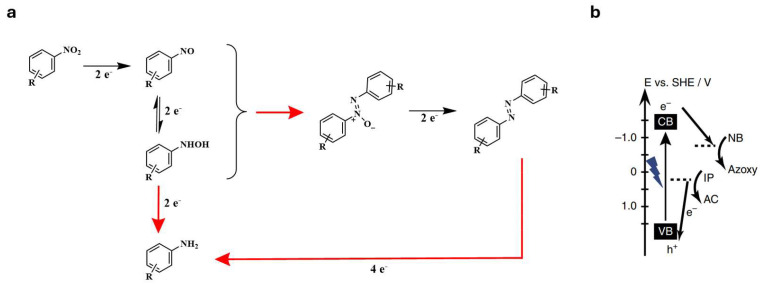
Photochemical process for reduction of nitrobenzenes to azobenzenes (**a**) and energy diagram of this conversion (**b**). Figure (**b**) reprinted with permission from Ref. [88] under Creative Commons CCBY license.

In addition, azobenzenes can be prepared via the photochemically excited reduction of nitrobenzenes (Figure 16) [88].

In the reaction shown above, the inexpensive graphitic C_3_N_4_ represents an efficient photocatalyst for the selective syntheses of a series of azo- and azoxy-aromatic compounds from their corresponding nitroaromatics when exposed to either purple (410 nm) or blue light (450 nm) excitation (Figure 16a). As can be seen in Figure 16b, the photoexcited e^−^ in the conduction band (CB) of g-C_3_N_4_ possesses sufficient energy to initiate the nitrobenzene reduction. Concurrently, isopropanol (IP) acts as a sacrificial electron donor, undergoing oxidation to acetone (AC), which generates two protons and releases two electrons that recombine with the holes (h^+^) in the valence band (VB).

The high efficiency and high selectivity towards azo- and azoxy-aromatic compounds can be attributed to the weakly bound photogenerated surface-adsorbed H-atoms and a favourable N-N coupling reaction. The selectivity in this type of reaction is shown by the low proportion of amine formed during the azobenzene synthesis reaction (up to 10%), irrespective of the functional groups present on the nitrobenzene. In addition, using 410 nm light, selective conversion to azobenzene and, at 450 nm, selective conversion to azoxybenzenes occur. The yields of the reactions are very high (between 90% and 100%) and do not significantly depend on the functional group on the nitrobenzene [88].

## 4. Complexation and Crystal Structures

**Figure 17:**
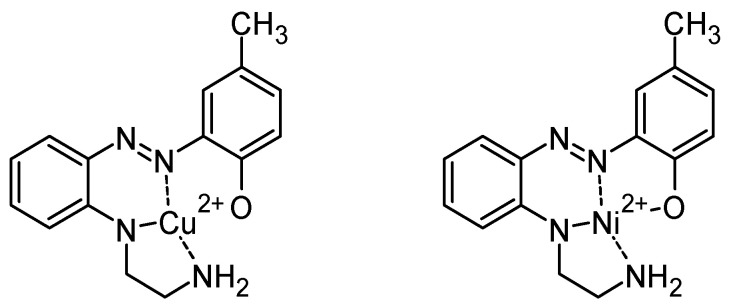
Example of complexes obtained in ref [89].

In 1969, Baer et al. studied metal complexes of azo dyes [89]. Particularly, they added cupric chloride or nickel chloride in an aqueous solution of ethylenediamine to o-halide-o’-hydroxyazo compounds, and they obtained several compounds (Figure 17) analyzed by their melting point, their empirical formula, and also their λ_max_ value (nm). Their magnetic susceptibility was also determined. For example, the nickel complex was found to be diamagnetic, indicating that the complex has a square planar geometry around the nickel atom. But the copper complexes examined had a magnetic moment equal to or greater than the spin-only value of 1.73, suggesting that no significant interactions occur between copper atoms. The authors indicated that X-ray would have been required to establish the stereochemistry with certainty.

With this in mind, a relevant example is the complex chlorido[(E)-3-hydroxy-2-methyl-6-(quinolin-8-yldiazenyl)phenolato]copper(II) monohydrate [90]. In this compound, the hydroxyazobenzene ligand is deprotonated at the ortho position and coordinated to the Cu(II) centre, forming a pseudo-square planar geometry. The crystal structure was determined by X-ray diffraction, revealing the following characteristic bond lengths:

- Cu–O: 1.917 Å.
- Cu–N: (quinoline): 2.008 Å.
- Cu–N: (azo): 1.945 Å.
- Cu–Cl: 2.280 Å.

**Figure 18:**
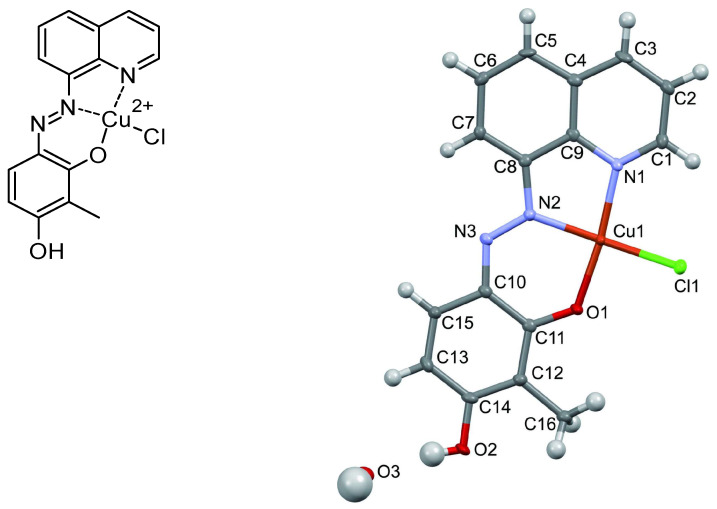
Copper complex and X-ray structure of chlorido[(E)-3-hydroxy-2-methyl-6-(quinolin-8-yldiazenyl)phenolato]copper(II) monohydrate. Reprinted with permission from Ref. [90]. Copyright 2022, IUCr Journals.

The complex also shows a weak axial coordination between copper and a chlorine atom from a neighbouring molecule, with a Cu–Cl distance of 2.739 Å, suggesting an intermolecular interaction (Figure 18).

**Figure 19:**
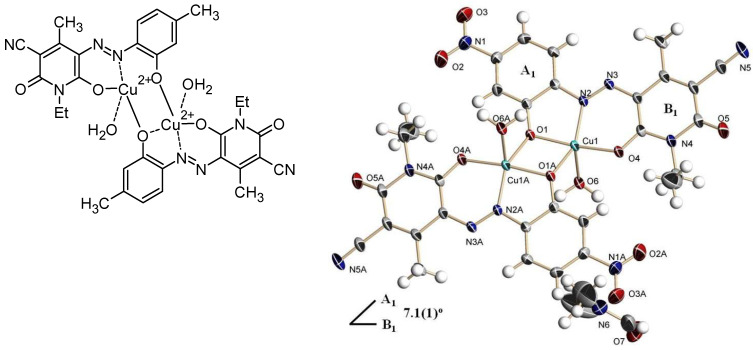
Copper complex and X-ray structure of intermediate. Reprinted with permission from Ref. [91]. Copyright 2017, Elsevier.

Sometimes, complexation occurs within a synthetic pathway to obtain, after demetallization, ortho-OH azoaryl dyes. This was the case in 2017 where a dinuclear copper (II) complex was synthesized from copper diacetate and a hydrazone molecule by a Fenton reaction using H_2_O_2_ [91]. The crystal structure of this intermediate was determined in DMF by X-ray diffraction, revealing the following characteristic bond lengths (Figure 19):

- Cu1–O1: 1.944 Å.
- Cu1–O1A: 2.061 Å.
- Cu1–O4: 1.920 Å.
- Cu1–O6: 2.140 Å.
- Cu1–N2: 1.925 Å.

**Figure 20:**
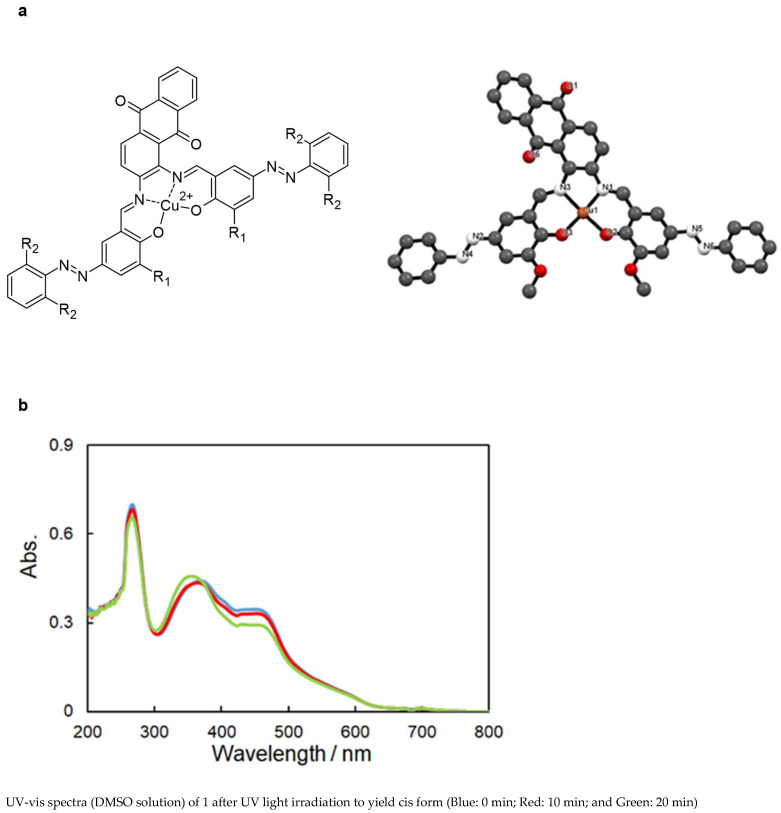
(**a**) Structures and (**b**) photoisomerization of example complex. Article is open access and distributed under terms and conditions of CC BY 4.0 licences [92].

Schiff bases and especially (chiral) salen metal complexes can been employed in (asymmetric) catalysis because of their advantages in stereochemical tuning (with respect to chirality) and redox properties with proper metal ions [92]. These molecules (Figure 20) were synthesized and gave the following crystal structure:

Example for R_1_ = OCH_3_, R_2_ = H:

- Cu1–O2: 1.854 Å.
- Cu1–O3: 1.885 Å.
- Cu1–N1: 1.957 Å.
- Cu1–N3: 1.913 Å.

Concerning photoisomerization capability, the change in UV-vis spectra due to the *trans*-to-*cis* photoisomerization of the azobenzene moiety (R_1_ = OCH_3_; R_2_ = H) showed a shift in π-π* bands to a shorter wavelength. The stable *trans* form was converted to the *cis* form by a metal-to-ligand charge transfer (MLCT) transition, where electrons move to the ligand, as well as other transitions. Moreover, the reversible *cis*-*trans* photoisomerization, which converts the *cis* to the *trans* form by visible light irradiation, was observed for all complexes (Figure 20).

**Figure 21:**
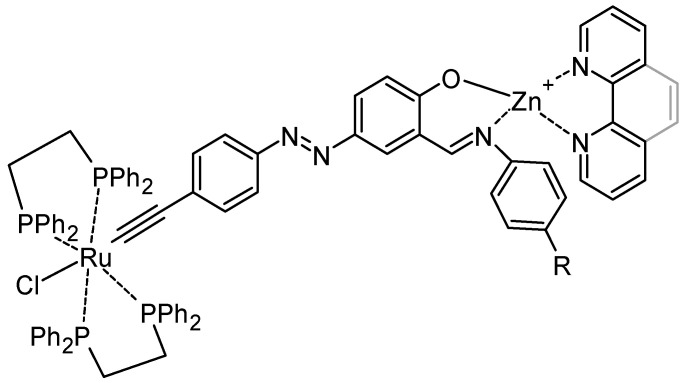
Azo bi-metallic complex.

Bi-metallic compounds can also be obtained by the reaction of an ethyynylazobenzene and [RuCl_2_(dppe)_2_]. This intermediate can react with anilines to obtain Schiff bases, and by the reaction of zinc salts in the presence of phenanthroline or bipyridine, complexes are formed (Figure 21) [93]. It is noticeable that luminescence is observed for all complexes as a result of the intra-ligand π→π* transition and chelation enhanced fluorescence (CHEF) by the coordination of the imine group to Zn(II) in the complexes. The presence of azo chromophore with extended π-conjugation enhances the emission properties of the complexes and varies considerably with donor–acceptor substituents and the π-acidic character of the co-ligands.

## 5. Applications of Phenolic Azobenzene Complexes

### 5.1. Colorimetric Sensors

**Figure 22:**
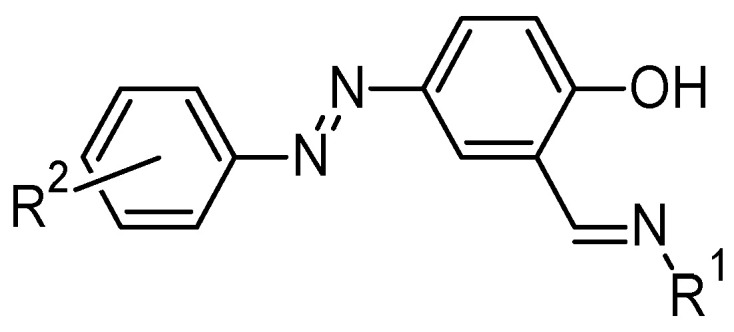
General structure of azo–Schiff bases.

Hydroxyazobenzene compounds are a class of azo dyes known for their vivid colouration. The hydroxyl group plays a key role in metal ion binding through coordination chemistry, especially when the azo-OH moiety is in the *ortho* position of a Schiff base (Figure 22) [94]. When these molecules interact with metal cations, they form stable chelate complexes. These complexes alter the molecule’s electron distribution, leading to a visible colour change. This property is exploited in colorimetric detection methods for various cations. UV-Vis spectrophotometry can quantify these changes based on absorbance shifts. Each metal ion produces a characteristic spectral change, enabling selective detection. Commonly detected cations include Cu^2+^ and Cr^3+^ [95], Cd^2+^ and Hg^2+^ [96], Co^2+^, and Al^3+^ [97,98]. Detection is often pH-dependent as protonation affects the ligand binding sites. The simplicity of visual detection makes them suitable for rapid, low-cost analysis [94].

**Figure 23:**
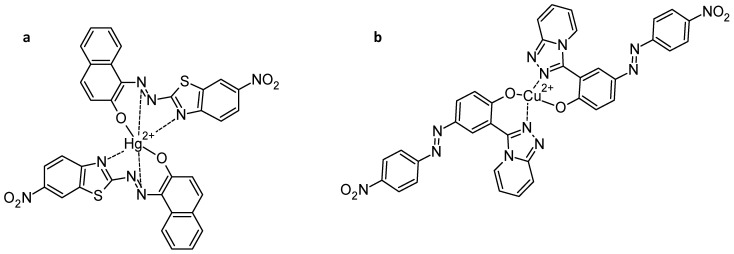
Azo phenol-based probe for (**a**) Hg^2+^ detection [102] and (**b**) Cu^2+^ detection [103].

In the domain of pollutant removal, it is also important to be able to perform selective cation detection [99,100,101]. With this in mind, azo-phenol-based receptors with an azo core linked to naphthol and variously substituted benzothiazole units were synthesized and investigated for their optical properties. Among them, the nitro-substituted derivative showed enhanced fluorescence upon interaction with Hg^2+^ ions, which enabled detection with the “naked eye“ [102]. Spectroscopic analyses—including UV-Vis absorption, fluorescence emission, FT-IR, and ^1^H NMR—revealed that the Hg^2+^ ion coordinates favourably with the phenolic oxygen, the azo nitrogen adjacent to the naphthol ring, and the thiazole nitrogen of the benzothiazole unit. This interaction occurs in a terdentate fashion (Figure 23). In addition, photoinduced and Hg^2+^-induced proton transfer reactions supported the presence of *cis*-*trans* isomerism and azo-hydrazone tautomerism in these systems.

**Figure 24:**
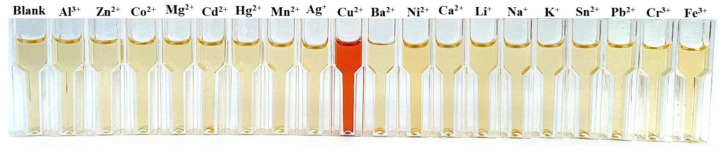
Selective chemosensing of copper cations. Reprinted with permission from Ref. [103]. Copyright 2025, Elsevier.

Only recently (2025) was a naked-eye chemosensor developed that makes it possible to quickly and selectively detect copper cations in solution (Figure 24) [104].

### 5.2. Smart Materials and DSSCs

Hydroxyazobenzene compounds exhibit promising potential as dyes for dye-sensitized solar cells (DSSCs) due to their ability to form a complex with metal cations. The hydroxyl groups, mainly supported by the Schiff base in their structure, facilitate coordination with metal ions such as Ti^4+^, Cu^2+^ [103,105], Fe^2+^ [106], or Zn^2+^ [106], which enhances electronic communication with semiconductor surfaces.

**Figure 25:**
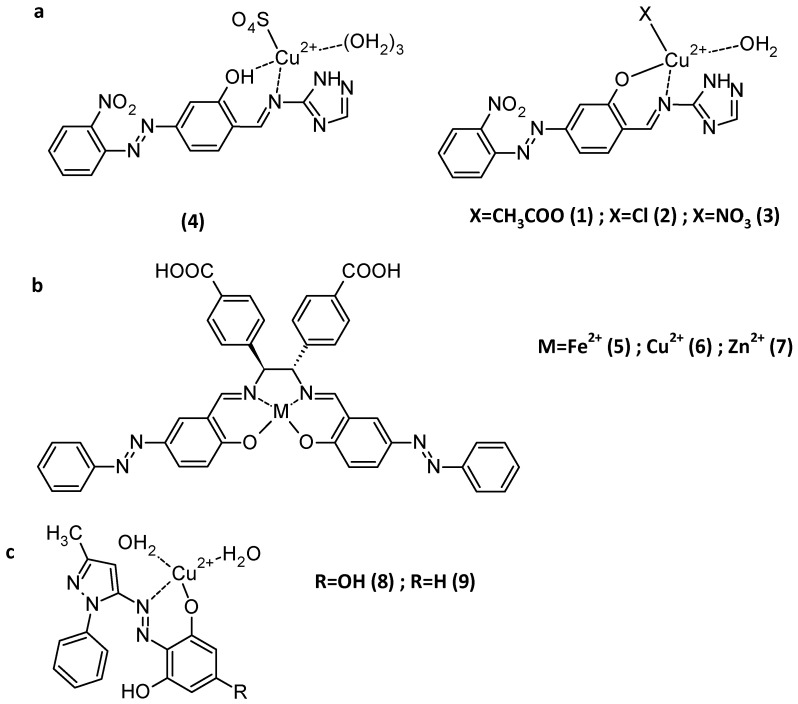
Molecules involved in DSSC applications (**a**) from ref. [103]; (**b**) from ref. [106]; (**c**) from ref. [106].

This complexation often results in improved light-harvesting capabilities by extending the absorption range into the visible spectrum (Figure 25, Table 4). The resulting metal–dye complexes can also promote efficient charge separation and electron injection into the conduction band of TiO_2_. Moreover, hydroxyazobenzene dyes are synthetically tuneable, allowing for the optimization of photophysical and electrochemical properties. Indeed, their structural versatility supports the fine-tuning of absorption maxima and redox potentials as azo dyes can be synthesized with various functional groups with different absorption spectra, dipole moments, and conjugation lengths [107]. 

Additionally, these complexes can exhibit strong anchoring to TiO_2_ surfaces [106]. As the hydroxyl group is complexed with a metal cation, it does not interfere with the other anchoring groups, as carboxylate, sulfonate, or pyridyl anchors, knowing that amongst these groups, OH yields the worst performance, with its inferior UV/vis absorption intensity on the TiO_2_ substrate and the lowest DSSC performance conversion efficiency [108].

**Table 4:** Spectral and electrochemical properties of compounds 1–9.

	**E_HOMO_ (eV)**	**E_LUMO_ (eV)**	**Δ** **E (eV)**	**Absorption Range/** **λ** **max (nm)**	**Reference**
1	−5.85	−3.13	2.72	387–679	[103]
2	−5.74	−3.11	2.73	440–727	[103]
3	−5.92	−3.18	2.74	472–752	[103]
4	−6.64	−4.02	2.62	511–790	[103]
5	−5.483	−3.429	2.234	368	[106]
6	−5.312	−3.785	1.527	387	[106]
7	−5.579	−3.279	2.282	352	[106]
8	−4.36	−3.73	0.63	450	[105]
9	−4.36	−3.83	0.53	402	[105]

Dye-sensitized solar cells (DSSCs) were constructed to evaluate the performance of hydroxyazobenzene molecules that complex cations under controlled laboratory conditions. These cells served as a testing platform to investigate the photoelectrochemical properties and potential of azobenzene-based dyes as light-harvesting materials. By producing DSSCs with these compounds, the aim was to gain insight into their efficiency, stability, and suitability for solar energy conversion applications.

**Figure 26:**
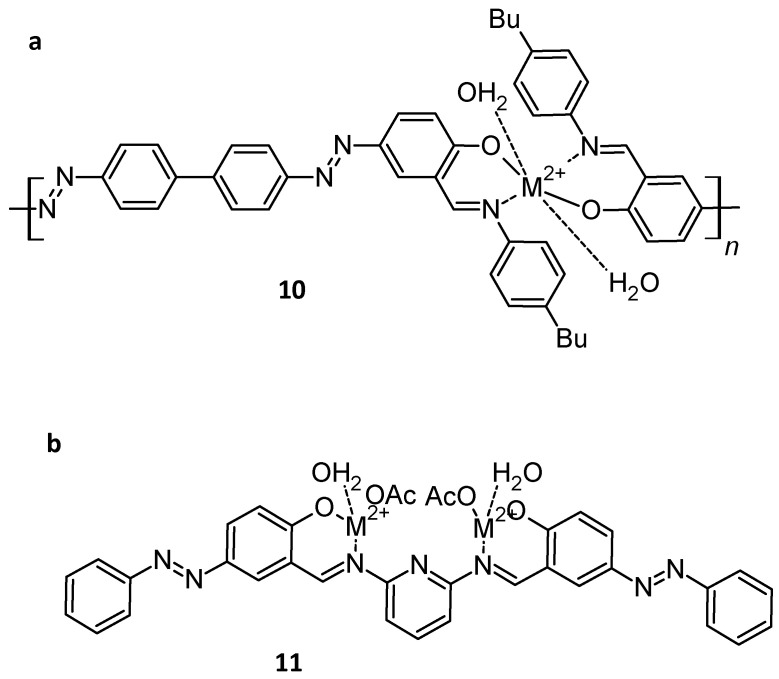
Complexes from (**a**) ref. [109] and (**b**) ref. [110].

For example, two types of materials were tested (Figure 26). The first one was a polychelate [109], whereas the second one was a metal complex [110].

The photovoltaic parameters of these devices were determined (Table 5). Open-circuit voltage (V_OC_) is the maximum voltage a solar cell can provide to an external circuit, which is derived from the splitting of hole and electron quasi-Fermi levels. In organic materials (OSC), disorder induces gap tail states. The relaxation of carriers into these tail states brings the electron quasi-Fermi level down and the hole quasi-Fermi level up and hence reduces V_OC_ [111]. In this context, compared to the reference N3 dye ([Ru(dcbpy)_2_(NCS)_2_]) [112], organometallic compounds 10 and 11 seem to have lower efficiency, but the results can vary a lot depending on the device used (full commercial device, light power, etc.). 

**Table 5:** Photovoltaic parameters with Voc: open-circuit voltage; FF: Fill factor; η: overall energy conversion yield.

Compound	**V_OC_, mV**	**FF**	**η, %**	
I_3_^−^/I^−^	724	0.70	8.0	[113]
N_3_	678	0.607	7.3	[112]
10				[109]
Cu_2+_	400	0.54	2.05	
11				[110]
Co_2+_	515	0.585	0.742	
Ni_2+_	495	0.689	0.328	
Pd_2+_	495	0.654	0.856	

### 5.3. Catalysis and Coordination Chemistry

**Figure 27:**
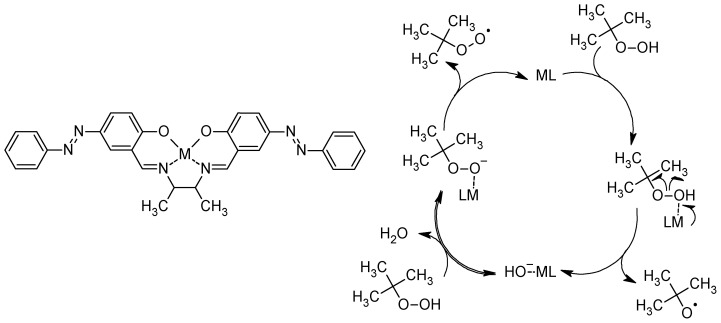
Azo–Schiff base catalyst and proposed mechanism of oxidant activation [114].

The catalytic activity of Cu(II), Co(II), and Ni(II) azo–Schiff base complexes (Figure 27) was tested for the oxidation of various alkenes (cyclooctene, cyclohexene, styrene, α-methylstyrene, and norbornene). It was found that under the optimized reaction conditions, the copper(II) complex displayed 94% conversion for the oxidation of cyclooctene, and the cobalt(II) and nickel(II) complexes exhibited 90 and 85% conversions for the oxidation of α-methyl styrene, respectively. The authors demonstrated that back electron transfer from the complexes to TBHP (tert-butyl hydroperoxide, the oxidant used) breaks the O-O bond and forms tert-butoxyl radicals [114].

**Figure 28:**
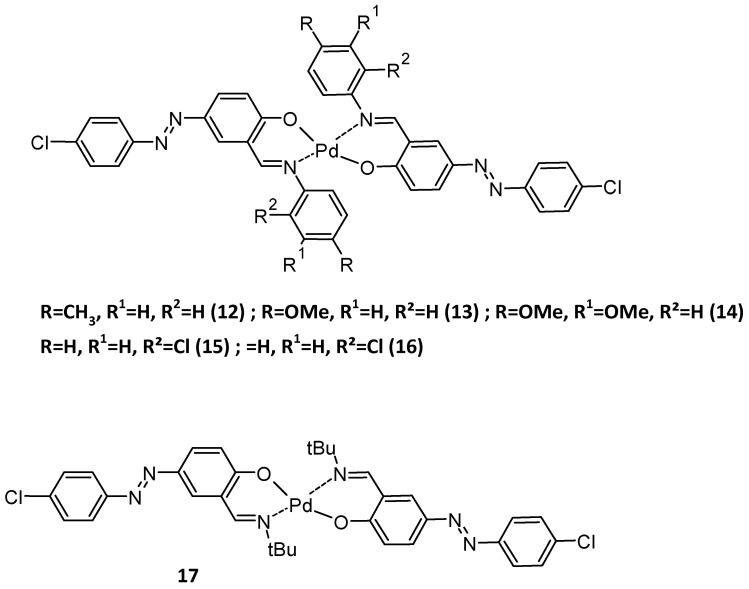
Some of the complexes used for Suzuki coupling [115].

Very recently, the Suzuki–Miyaura coupling reactions of phenylboronic acid with aryl bromides were also investigated using Pd(II) complexes (Figure 28) [115].

Various aryl bromides were tested with these complexes, giving very high yields (Table 6), and in the case where 4-bromoacetophenone was used, the highest yield of 99% was obtained with catalysts 12–14, while the lowest efficiency was obtained when catalyst 17 was used (94%). But in the presence of 4-bromobenzaldehyde, the highest yields were obtained with catalyst 15, while the lowest yield was observed when catalyst 14 was used with 86% efficiency. When an electron-donating group is present, catalysts 13 and 14 seem to work the best. So, there is not really any structure–activity relationship.

**Table 6:** Results of Suzuki–Miyaura coupling reactions [115].

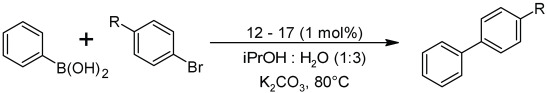		
R	Catalyst	Yield (%)
CH_3_	12	95
	13	88
	14	97
	15	91
	16	95
	17	90
OMe	12	96
	13	98
	14	98
	15	96
	16	93
	17	97
C(O)CH_3_	12	99
	13	99
	14	99
	15	97
	16	98
	17	94
CHO	12	94
	13	92
	14	86
	15	95
	16	94
	17	94

**Figure 29:**
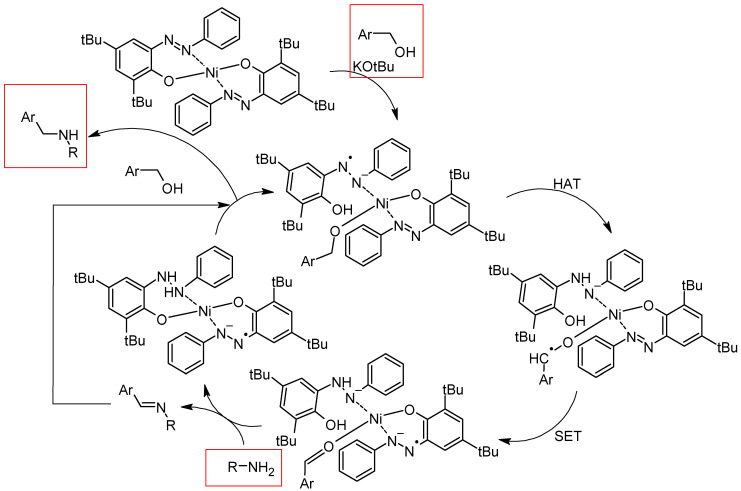
Proposed mechanism [116].

For the N-alkylation reaction via hydrogen borrowing, nickel catalysis using hydroxyazobenzene as a ligand was performed and gave access to the N-alkylation of a variety of anilines with alcohols, with moderate to good yields. What is interesting is that the hydrogen atom transfer (HAT, Figure 29) to the reduced azo backbone for alcohol oxidation occurred, as opposed to a conventional mechanism [116].

Bis(phenoxy-azo)titanium(IV) complexes were also used for the polymerization of styrene. It is interesting to note in this article that due to its poor σ-donating character, the N atom of the azo group forms long Ti−N bonds, which in turn lead to a more open coordination sphere around the Ti centre. Unfortunately, the bis(phenoxy-azo)titanium complexes do not undergo photoinduced E → Z isomerization in their chelating azobenzene ligand frameworks. We attribute this behaviour to the relatively strong Ti−N and Ti−O bonds, which cannot be broken in the course of the photoisomerization reaction [117].

When the diazo moieties are part of a porous organic polymer (POP), it can not only permit the complexation of metals such as zinc but also, with a high surface area, allow for the conversion of carbon dioxide. This is the case of Zn/HAzo-POP, which was able to catalyze the reaction between propylene oxide and CO_2_ [118].

### 5.4. Biomedical and Environmental Applications

The MCF-7 cell line is a widely used human breast cancer model, originally isolated from a metastatic breast cancer patient in 1970. These cells are estrogen receptor-positive (ER^+^) and progesterone receptor-positive (PR^+^), making them an ideal system for studying hormone-dependent breast cancer [119]. MCF-7 cells exhibit an epithelial-like morphology and grow in response to estrogen, allowing researchers to investigate the mechanisms of cancer proliferation, hormone signalling, and drug sensitivity [120]. HeLa cells are the first immortal human cell line, derived in 1951 from the cervical cancer tumour of Henrietta Lacks [121]. These cells are remarkable for their ability to proliferate indefinitely under suitable laboratory conditions, making them a cornerstone of biomedical research. HeLa cells have been used in countless scientific breakthroughs, including the development of the polio vaccine, cancer research, and studies on viruses and cell biology. However, they are highly genetically unstable, which can affect experimental outcomes. Despite ethical concerns about their origin, HeLa cells remain one of the most important tools in medical and biological research [122].

**Figure 30:**
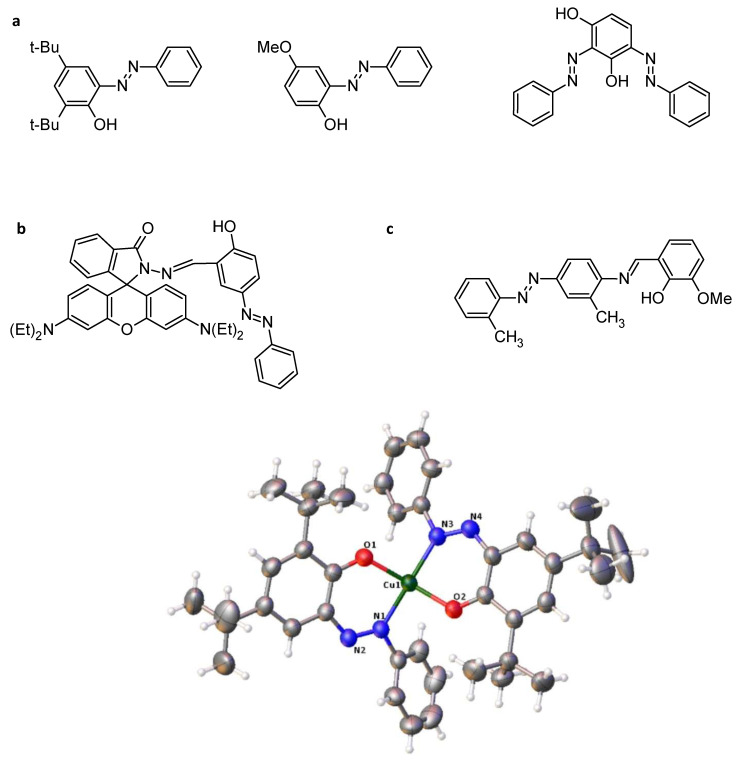
Structures of ligands (**a**) in ref. [123], (**b**) in ref. [124], and (**c**) in ref. [125] and ellipsoid representation of copper complex from (**a**) [123]. Reprinted with permission from Ref. [123]. Copyright (Elsevier) 2025.

In this context, it is urgently needed to find anticancer agents and especially cancer-selective ones. Very recently, the cytotoxicity of functionalized hydroxyazobenzene copper(II) complexes (Figure 30) was investigated in the human breast cancer cell line MCF-7 and the human cervical cancer cell line HeLa, demonstrating selective cytotoxicity against cancer cells with a GI_50_ < 10 µg/mL [123].

Another application, in which hydroxyazobenzene is coupled to rhodamine, is the fluorescence imaging of HeLa cells incubated with Cu^2+^. This experiment confirms that this type of sensor has good cell permeability and can be useful and specific for Cu(II) sensing in living cells [124].

In 2024, *o*-aminoazotoluene, which is capable of photoisomerization, and *o*-vanillin, a potent comutagen, were used to synthesize a new ligand which, upon complexation with copper(II), results in a new copper(II) complex—Cu(L)_2_ [125]. This complex had excellent results against MCF-7 cell lines with an IC50 of 4.2 ± 0.53 µM (Figure 30).

**Figure 31:**
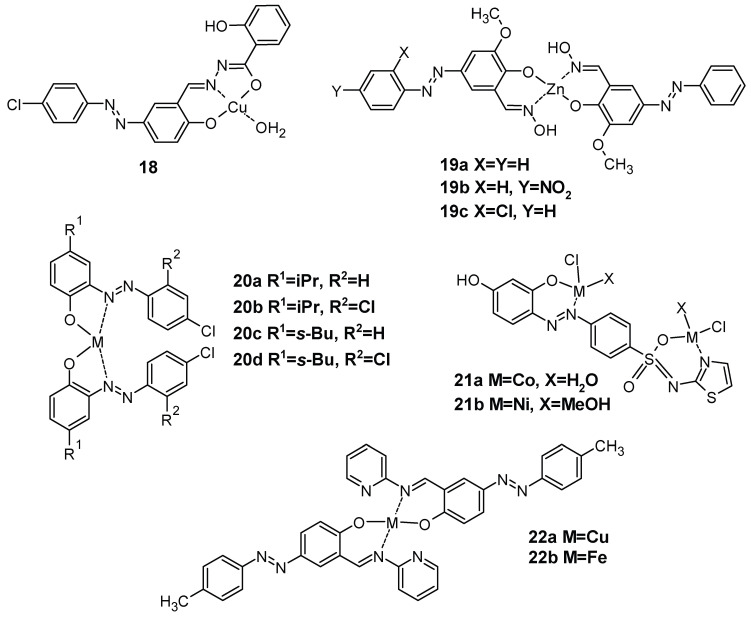
Structure of azo–Schiff base copper complex 18 [126], zinc complex 19 [127], copper and nickel complexes 20 [128], cobalt and nickel complexes 21 [129], and copper and iron 22 [130].

In the field of antimicrobial agents, and especially against *Staphyllococcus aureus* or *Pseudomonas aeruginosa*, it was shown that the stronger effect of complexes against the ligand alone could be due to the chelation of ligands with metal ions, facilitating the permeability of the drug to the cell’s environment. For example, Schiff base azobenzenes complexed with cobalt, nickel, or copper ions (Figure 31) decrease the minimal inhibitory concentration (MIC) from approximately 350 mg/L for the ligand alone to less than 50 mg/L [126]. Zinc complexes can also have excellent MIC values against Gram-positive and Gram-negative bacteria, compared to chloramphenicol (Table 7, Figure 31) [127]. Copper and nickel complexes of the same ligands were also compared for their ZOI (zone of inhibition) against several bacteria, and the results show that depending on the ligand or the metal, the structure–activity relationship is quite impossible to obtain [128].

When the examined organisms were *Salmonella typhimurium* and Staphylococcus aureus, the results obtained showed that Ni(II) complex 21b (Table 7, Figure 31) displayed good activity towards S. aureus, within the 11.97 mm inhibition zone, whereas Co(II) complex 21a showed high efficiency contra *S. Typhimurium* within the 11.13 mm inhibition zone [129]. It is interesting to note that azo-azomethine complexes 22a and 22b exhibited moderate antimicrobial activity against bacterial strains, but this activity was insignificant against fungi, revealing a good selectivity against pathogens [130].

**Table 7:** Compiled antimicrobial effects against bacteria from complexes **19**–**22**.

Micro-Organisms		ZOI	MIC	Ref
*Salmonella typhimurium*	**19a**	-	125 µM/mL	[127]
	**19b**	-	250 µM/mL	[127]
	**19c**	-	125 µM/mL	[127]
	**21a**	11.13 mm	-	[129]
*Staphylococcus aureus*	**19a**	-	7.81 µM/mL	[127]
	**19b**	-	15.63 µM/mL	[127]
	**19c**	-	3.90 µM/mL	[127]
	**20a**	15 mm(Cu), 12 mm (Ni)	-	[128]
	**20b**	17 mm (Cu), 13 mm (Ni)	-	[128]
	**20c**	17 mm (Cu), 12 mm (Ni)	-	[128]
	**20d**	16 mm (Cu), 14 mm (Ni)	-	[128]
	**21b**	11.97 mm	-	[129]
	**22a**	13 mm	128 µg/mL	[130]
	**22b**	11 mm	256 µg/mL	[130]
*Escherichia coli*	**19a**	-	62.5 µM/mL	[127]
	**19b**	-	250 µM/mL	[127]
	**19c**	-	62.5 µM/mL	[127]
	**20a**	15 mm (Cu), 20 mm (Ni)	-	[128]
	**20b**	19 mm (Cu), 14 mm (Ni)	-	[128]
	**20c**	20 (Cu)	-	[128]
	**20d**	12 mm (Cu), 14 mm (Ni)	-	[128]
	**22a**	15 mm	64 µg/mL	[130]
	**22b**	17 mm	256 µg/mL	[130]
*Bacillus subtilis*	**19a**	-	7.81 µM/mL	[127]
	**19b**	-	31.25 µM/mL	[127]
	**19c**	-	7.81 µM/mL	[127]
*Enterococcus faecalis*	**20a**	12 mm (Cu), 14 mm (Ni)	-	[128]
	**20b**	18 mm (Cu), 15 mm (Ni)	-	[128]
	**20c**	14 mm (Cu)	-	[128]
	**20d**	17 mm (Cu), 17 mm (Ni)	-	[128]
*Enterobacter cloacae*	**20a**	12 mm (Cu), 22 mm (Ni)	-	[128]
	**20b**	15 mm (Cu), 15 mm (Ni)	-	[128]
	**20c**	17 mm (Cu), 12 mm (Ni)	-	[128]
	**20d**	13 mm (Cu), 16 mm (Ni)	-	[128]
*Micrococcus luteus*	**20a**	13 mm (Cu), 14 mm (Ni)	-	[128]
	**20b**	21 mm (Cu), 17 mm (Ni)	-	[128]
	**20c**	19 mm (Cu), 14 mm (Ni)	-	[128]
	**20d**	18 mm (Cu), 16 mm (Ni)	-	[128]

## 6. Conclusions

Phenolic azobenzene derivatives represent a uniquely versatile class of compounds at the intersection of photochemistry, coordination chemistry, and materials science. Their dual functional nature—combining the reversible photoisomerization capability of the azobenzene core with the metal-binding propensity of the phenolic group—enables a wide range of applications, from colorimetric sensing and smart materials to catalysis, solar energy conversion, and biomedical fields. The diversity in synthetic strategies, including azo coupling, Baeyer–Mills reactions, and oxidation–reduction pathways, allows for fine structural tuning and functionalization, further broadening their applicability.

Particularly notable is their effectiveness as ligands in the formation of stable metal complexes, which exhibit enhanced photophysical, catalytic, and bioactive properties. These complexes have shown promise not only in environmental and industrial contexts but also in the development of anticancer and antimicrobial agents. As demonstrated in dye-sensitized solar cells (DSSCs), phenolic azobenzene–metal complexes contribute to improved light-harvesting and electron transfer efficiencies. 

Future developments in this field could focus on expanding the functional diversity of these ligands, improving synthetic efficiencies, and deepening the understanding of structure–activity relationships to design next-generation materials with tailored properties. Overall, phenolic azobenzenes stand out as highly adaptable and functional scaffolds for advancing molecular design across multiple scientific domains.

## Figures and Tables

**Table 1:** Main properties of two parts of hydroxyazobenzenes.

Property	Azobenzenes	Phenolic Compounds
Acid or Base Behaviour	Stable but protonated under strong acid conditions	Weak acids, form phenoxide anions in base conditions
Electronic Effects	Azo (-N=N-) bond conjugation influences absorption	-OH is electron-donating, affects reactivity
Photoisomerization	UV–visible light-induced *trans*-*cis* switching	No isomerization, but light can degrade phenols
Redox Chemistry	Reduced to hydrazo (-NH-NH-) under mild conditions	Oxidized to quinones in oxidative media
Electrophilic Substitution	Substituent-dependent reactivity	Highly activated towards substitution reactions
Metal Coordination	Forms transition metal complexes	Coordinates metal ions (e.g., Fe^3+^, Cu^2+^)

**Table 2:** Synthesis of phenolic azobenzenes using diazonium salt and NaOAc as base.

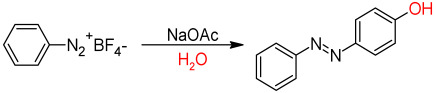	
Solvent	Yield [%]
Methanol	46
Ethanol	42
Acetonitrile	40
Acetone	41
Dichloromethane	0
Methanol–H_2_O = 1:1	80
Methanol–H_2_O = 3:1	89
Methanol–H_2_O = 9:1	63

**Table 3:** The effects of the substituent on aniline on the formation of azobenzene.

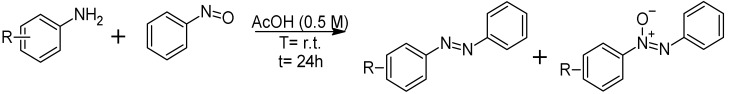		
R	Azobenzene yield [%]	Azoxybenzene yield [%]
*p*-NMe_2_	34	45
*p*-NH_2_	28	43
*o*-NH_2_	8	91
*p*-OMe	≥95	5
2,6-dimethoxy	46	35
*p*-NHCO_2_^t^Bu	87	6
*p*-OH	34	69
*o*-OMe	82	13
*p*-Me	95	≤5
*o*-NHCO_2_^t^Bu	85	12
*o*-Et	77	20
*p*-I	≥95	≤5
H	≥95	≤5
*o*-Br	49	8
*p*-CO_2_Me	82	6
2,6-difluoro	12	≤5
*p*-CN	64	7
*p*-CF_3_	≥95	≤5
*o*-NO_2_	≤5	≤5
*p*-NO_2_	19	≤5
